# Rhabdomyolysis following Cardiac Surgery: A Prospective, Descriptive, Single-Center Study

**DOI:** 10.1155/2016/7497936

**Published:** 2016-02-29

**Authors:** Amr S. Omar, Hesham Ewila, Sameh Aboulnaga, Alejandro Kohn Tuli, Rajvir Singh

**Affiliations:** ^1^Department of Cardiothoracic Surgery/Cardiac Anaesthesia & ICU Section, Heart Hospital, Hamad Medical Corporation, P.O. Box 3050, Doha, Qatar; ^2^Department of Critical Care Medicine, Beni-Suef University, P.O. Box 62511, Beni-Suef, Egypt; ^3^Department of Anesthesia, Suez Canal University, P.O. Box 351-41511, Ismailia, Egypt; ^4^Department of Anesthesia, Ain Shams University, Kasr-El-Zaafaran, P.O. Box 11566, Cairo, Egypt; ^5^Department of Cardiology Research Centre, Hamad Medical Corporation, P.O. Box 3050, Doha, Qatar

## Abstract

*Purpose*. Rhabdomyolysis (RML) following cardiac surgery and its relationship with acute kidney injury (AKI) require investigation.* Patients and Methods*. All patients undergoing cardiac surgery in our hospital were enrolled in this prospective study during a 1-year period. To investigate the occurrence of RML and its association with AKI, all patients in the study underwent serial assessment of serum creatine kinase (CK) and myoglobin levels. Serial renal function, prior statin treatment, and outcome variables were recorded.* Results*. In total, 201 patients were included in the study: 185 men and 16 women with a mean age of 52.0 ± 12.4 years. According to the presence of RML (CK of ≥2,500 U/L), the patients were divided into Group I (RML present in 17 patients) and Group II (RML absent in 184 patients). Seven patients in Group I had AKI (41%) where 34 patients in group II had AKI (18.4%), *P* = 0.025. We observed a significantly longer duration of ventilation, length of stay in the ICU, and hospitalization in Group I (*P* < 0.001 for all observations).* Conclusions*. An early elevation of serum CK above 2500 U/L postoperatively in high-risk cardiac surgery could be used to diagnose RML that may predict the concomitance of early AKI.

## 1. Introduction

Muscle necrosis and subsequent release of intracellular muscular constituents in the circulation are characteristic of rhabdomyolysis (RML). Asymptomatic elevation of muscle enzymes may be the only manifestation of RML. However, extreme cases may be associated with marked elevation of these enzymes, electrolyte disturbances, and development of acute kidney injury (AKI). Bywaters and Beall were the first to report RML in 1941, when four victims of crush injuries died after 1 week of developing AKI. An autopsy study revealed pigmented casts in the renal tubules, but the association of muscle injury and renal failure was unexplained [1].

Immediate circumstances may precede RML, such as surgical trauma, postictal state, or extraordinary physical exertion. However, in some cases no precipitating factors of RML are found. Inherited enzymatic deficiencies, electrolyte disturbances, infections, toxins, drugs, and endocrinopathies could be possible causes of RML [2]. RML can be a complication of bariatric surgeries [3]. The incidence of RML after cardiac surgery is still unclear, although cases of RML after cardiac surgery have been reported [4]. Some individual case reports have mentioned a severe form of RML after coronary artery bypass grafting (CABG) with renal failure that required hemodialysis for 40 days [5].

Clinical and biochemical consequences following RML could lead to compartment syndrome, or even death. Excessive weight in obese patients may act as compressive pressure and is recognized as a risk factor of RML [6]. Increased serum myoglobin levels after cardiac surgery are linked to increased mortality and the need for renal replacement therapy [7]. Myoglobin is a low-molecular-weight heme protein, which is plentiful in cardiac and skeletal muscles. Myoglobin is rapidly released from necrotic muscle, while subsequent rapid renal clearance and high concentrations are associated with RML-induced renal failure [8].

Lagandré et al. [9] described precipitating factors that may lead to RML after bariatric surgery. These factors included obesity, prolonged supine postures, duration of the procedure when it is extended longer than 7 h in nonobese patients, and diabetes with concomitant microangiopathy and its possible metabolic complications, such as hypernatremia with hyperosmolarity, hypokalemia, hypocalcemia, and hypophosphatemia. Patients with American Society of Anesthesiologists (ASA) III and IV physical status are at higher risk of developing RML. Obese patients with ASA classification III and IV especially those with a body mass index > 55 kg/m^−2^ are at the highest ASA risk for RML [9].

The first signs and symptoms of RML are usually reported during the first 24 h after injury, although these may appear earlier. Suspicion of RML is usually based on clinical manifestations (reddish-brown urine, gluteal and back pain, and oliguria) and must be confirmed by laboratory studies. A fivefold elevation of serum creatine kinase (CK) levels (1050 U/L) is considered diagnostic for RML (males: CK > 1160 U/L; females: CK > 1075 U/L) [3, 10]. In cardiac surgery a higher set value to diagnose RML is described due to release of CK from related myocardial injury (2500 U/L) [11]. Severe RML is diagnosed when CK levels are higher than 10,000 U/L [12].

This study aimed to investigate the development of RML following cardiac surgery and its association with AKI. We also examined perioperative risk factors that may facilitate the occurrence of RML in cardiac surgery and the consequences of RML in this setting.

## 2. Methods

We performed a prospective, descriptive, single-center study with purposive sampling that examined the occurrence of RML and its association with AKI. All of the patients in the study underwent serial assessment of serum CK and myoglobin levels from baseline in the postoperative period. The study was conducted from February 2013 to February 2014 over 12 months in the cardiothoracic intensive care unit, Hamad Medical Corporation (12 beds). Approval for the study was obtained from the ethical committee (reference number 13001/13). Informed consent was waived for all patients by the Hamad Medical Corporation ethics committee because no specific intervention was carried out and sampling was part of routine care to make an early diagnosis of RML. In our patients, baseline muscular injury markers (CK, myoglobin, CK-MB isoform, and high-sensitivity troponin T) were measured, while two of these parameters (CK-MB isoform and high-sensitivity troponin T) were measured to quantify myocardial injury. Serum myoglobin levels were assessed by immunoassay from Beckman Coulter (Analis, Suarlée, Belgium). These laboratory markers were measured at the same time points by accredited hospital laboratory.

The following data were obtained: laboratory data that are routinely obtained in our intensive care unit (ICU) on admission; and demographic and clinical information, including age, sex, race, medical comorbidities, drugs, type of surgery, anesthesia time, cardiopulmonary bypass (CPB) time, aortic cross-clamp time, use of inotropes and vasopressors, Euro-SCORE, statin therapy, length of mechanical ventilation, and stay in the ICU and hospital. We systematically searched for risk factors for RML, as reported in previous studies. Complications and outcomes (AKI, arrhythmia, infection, stroke, need for dialysis, and mortality) were recorded for each patient. Patients with preexisting renal failure on dialysis or hepatic failure were excluded from the study. Once the diagnosis of RML was made, we used fluid loading and diuresis to treat our patients.

### 2.1. Study Definitions

Postcardiac surgery RML was suspected when serum CK levels were 2500 U/L or higher [11]. According to the consensus definition proposed by the Acute Kidney Injury Network, AKI was defined as an abrupt (within 48 h) reduction in kidney function, defined as an absolute increase in serum creatinine concentration of 0.3 mg/dL or greater (26.4 *μ*mol/L) or a percentage increase of 50% or greater (1.5-fold from baseline) [13]. Myoglobin was used to confirm diagnosis of RML [14]. Measuring the change in high sensitive troponin T is useful to quantify the extent of perioperative myocardial injury [15]. The European system for cardiac operative risk evaluation (Euro-SCORE) [16] was used to assess differences in patients' risk profiles, and the ASA classification was used to categorize the surgical risk [17].

### 2.2. Statistical Analysis

Results are presented as mean ± standard deviation for quantitative data and frequency and proportion for qualitative data. The data were analyzed to test for statistically significant differences between variants. For quantitative data, Student's *t*-test was used to compare two groups. For qualitative data, the chi-squared test was used and odds ratio was calculated. Multivariate regression analysis was performed for statistically significant data in the univariate analysis. Variables influencing RML in our and previous analyses were assessed by multivariate regression analysis. The primary data parameter for the study was defined as the peak CK level. Clinical and laboratory data were entered into a database (Microsoft Excel 2010; Microsoft Corporation, Redmond, WA, USA), and statistical analyses were performed using statistical software (SPSS, version 16; SPSS, Inc., Chicago, IL, USA).

## 3. Results

### 3.1. Clinical Variables in the Groups

**Figure 1:**
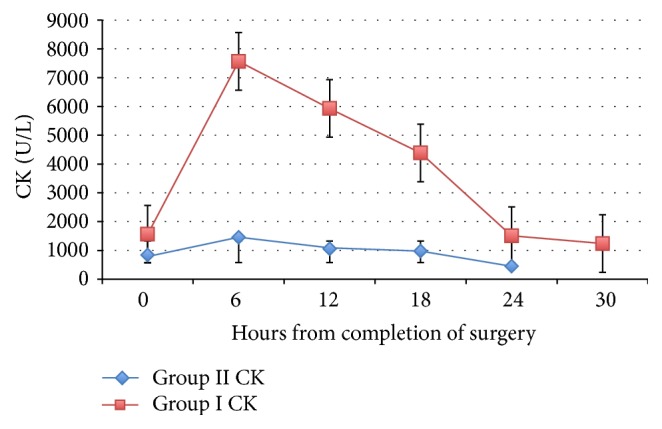
Dynamic changes in creatine kinase over time. The patients were divided into two groups: Group I (CK ≥ 2500 U/L) and Group II (CK < 2500 U/L). Postoperative changes in the CK level in both groups are shown above (0-hour sampling immediately after surgery). Error bars: ±SD.

Two hundred and one patients were enrolled in our study, with a mean age of 52.0 ± 12.4 years. Patients were divided into two groups according to the peak level of CK. Group I had a CK level equal to or higher than 2500 U/L; Group II had CK levels less than 2500 U/L. The dynamic changes in CK over time were noted in Figure 1. Seventeen out of the 201 patients (8.4%) developed RML according to our cutoff point. Both groups were matched regarding age and sex (Table 1). No significant difference in hypertension or diabetes, whether insulin-dependent or non-insulin-dependent, was found between both groups. Preexisting chronic renal disease or liver disease was not different between the groups. Ethnic predisposition was not associated with prevalence of RML. Among stating users RML was lower in Group I than Group II (47.1 versus 73.5%). There was no significant difference between the groups who underwent CABG. For patients who had valvular surgery, fewer patients had RML in Group I (11.7%) than in Group II (21.7%). All of the patients who entered the ICU after surgery for aortic dissection developed RML (three patients). The urgency of the procedure was not associated with RML. Dopamine was used more in Group I (62%) than in Group II (25%, *P* = 0.03). Preexisting heart failure or use of intra-aortic balloon pump (IABP) was not associated with a higher incidence of RML. Infectious complications were significantly higher in Group I (11.8%) compared with Group II (2.2%, *P* = 0.05), while other complications (arrhythmia and myocardial infarction) were not different between the groups.

### 3.2. Laboratory and Prognostic Variables in the Groups

Baseline creatinine levels were not significantly different between the groups (Table 2, 95.6 ± 35.3 *μ*mol/L in Group I versus 92.7 ± 61 *μ*mol/L in Group II, *P* = 0.8). Patients with RML showed a significant association with AKI (41% in Group I versus 18% in Group II, *P* = 0.02), and two patients required regular hemodialysis. High-sensitivity troponin T and CK-MB levels were significantly higher in Group I than in Group II (3606 ± 110 ng/L versus 1064 ± 81 ng/L and 83.4 ± 26 U/L versus 40.3 ± 3.1 U/L, resp., both *P* = 0.001). Myoglobin levels were significantly higher in Group I than in Group II (1120 ± 250 ng/mL versus 450 ± 195 ng/mL, *P* = 0.0001). The length of stay in the ICU and in hospital, and the length of mechanical ventilation, were significantly higher in Group I than in Group II (all *P* = 0.001).

**Figure 2:**
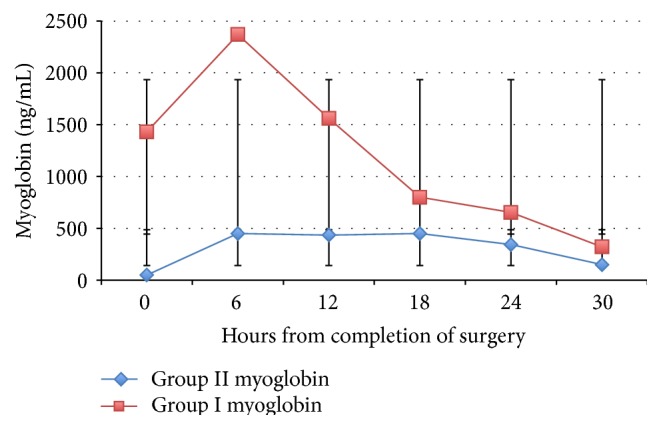
Dynamic changes in myoglobin in both groups in relation to time. The patients were divided into 2 groups: Group I (myoglobin ≥ 1000 ng/mL), and Group II (myoglobin < 1000 ng/mL). Postoperative changes in myoglobin both groups are shown above (0-hour sampling immediately after surgery). Error bars: ±SD.

**Figure 3:**
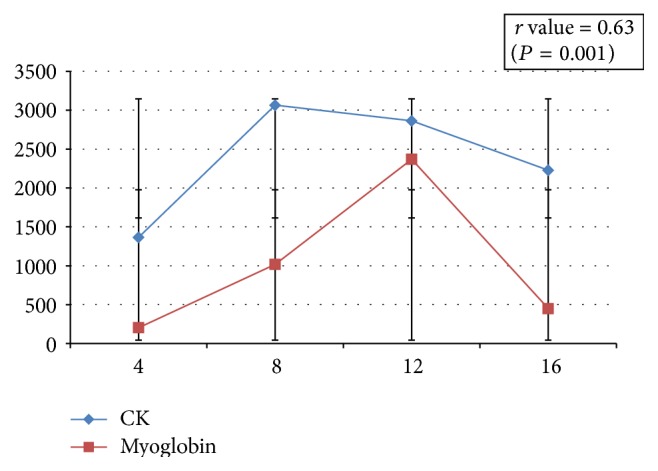
Relationship between CK and myoglobin.

Patients were divided again according to the myoglobin cutoff point of 1000 ng/mL. Dynamic changes in myoglobin in both groups in relation to time were noted in Figure 2. Three out of the 8 patients who had high myoglobin levels above 1000 ng/mL had higher incidence of AKI, and 38 out of 193 patients who had low myoglobin levels had AKI (37.5 versus 19.6%, *P* = 0.05). Myoglobin and CK levels tended to show an early rise, but CK longer than myoglobin (Figure 3). Hyperkalemia was encountered as an early warning sign because six out of 17 patients who developed RML had unexplained high potassium levels at an early stage in the first 4 hours after surgery (35.2%). Multivariate analysis (Table 3) showed that high CK is likely to associate CABG surgery and dopamine usage (*P* = 0.03 and 0.037, resp.). Finally, within the AKI group (44 patients) we studied the value of cut point of myoglobin (1000 ng/mL); there was high association of AKI in the high myoglobin group but without statistical difference 37.5% versus 19.5% (*P* = 0.2). We found a relationship between CK and myoglobin levels in the form of a significant increase in the myoglobin level with the rise in the CK level *r* value = 0.63 (*P* = 0.001) (Figure 3 and Table 4).

## 4. Discussion

The incidence of RML after cardiac surgery remains unclear, and some authors have mentioned an incidence of 19% for RML after CABG, with a direct relation between AKI and RML [6]. Myocardial injury that is experienced after cardiac surgeries cannot completely explain the observed myoglobinemia [6]. Our study was designed to investigate the incidence of RML after cardiac surgery and its relation to AKI. In our study, 17 patients developed RML, with an incidence of 8.41%. Black et al. described racial variation in serum CK levels where they found that Afro-Caribbean persons have higher levels of CK than the Caucasian population [18]. Based on this finding, we compared Arabs and Asians regarding the association of RML but did not find any significant correlation (*P* = 0.4).

Numerous factors may raise the propensity of RML after cardiac surgery but it is relatively uncommon after CPB [19]. Direct femoral artery cannulation [20], arterial diseases, a long extracorporeal circulation, low cardiac output syndrome, and continuous epinephrine infusion have been described as participatory factors of RML. Moreover, diabetes mellitus, extremes of age, and preexisting renal diseases are also thought to be participatory factors of RML [21, 22]. Incorrect positioning during surgery with pressure necrosis has also been reported [23, 24] and patients with an IABP are associated with RML [20]. In our study, both groups were matched regarding age and sex. However, our study did not include extremes of age, and patients who had diabetes or hypertension were not associated with a high incidence of RML in our study. In addition, both groups were matched regarding the Euro-SCORE and BMI. Our patients generally had a low body mass index (27.8 ± 5.1). The use of an IABP was not associated with a higher incidence of RML, and only 10 (4.9%) patients in our study required this intervention. It might induce RML when it causes limb ischemia, which was encountered in two patients in Group II, but they did not have compartment syndrome.

Unusual positions and increased pressure in certain areas during anesthesia allow sustained high pressure on the muscles. This results in muscle ischemia, sarcolemma injury, sodium-potassium pump disruption, electrolyte imbalance, and failure of energy supply to muscle fibers [25].

Statin users were not associated with the development of RML in our study. In fact, we found a significantly lower incidence of RML among patients taking statins (*P* = 0.02). Some studies have shown that statins have a high association with perioperative mortality [26]. The advantages of statins on surgical mortality might outweigh the postoperative risks. Eventually, all CABG patients should receive lifelong statin therapy in the absence of contraindications [27]. Kulik and Ruel [28] emphasized that statin-associated RML is more pronounced in patients with certain metabolic abnormalities, whereas our study population exhibited a relatively lower mean age and number of metabolic complications. The mean length of ventilation in our study was 503 ± 405 min. During this time, propofol was used at a dose of less than 1 mg/kg/h. Propofol infusion syndrome with concomitant development of RML has been encountered with use of doses as a high as 5 mg/kg/h for more than 48 h [29]. We did not find a significant association with use of epinephrine or norepinephrine in the patients who developed RML. However, dopamine users were associated with more RML events (Tables 2 and 3). We found that patients who developed an early infection had a higher incidence of RML, but this did not reach statistical significance. Viral as well as bacterial infections have been claimed to cause RML [30].

The urgency of the procedure did not appear to be associated with RML. Patients who underwent CABG had a higher incidence of RML than those who underwent valvular surgeries in multivariate analysis. All of the patients who had aortic dissection surgeries developed RML. Benedetto et al. [6] reported a 40% incidence of RML after CABG, but the incidence of CABG in our study was lower. No previous studies have addressed the association of RML after valvular surgery. In our study, two patients developed RML after valvular surgery. RML could be a common complication after surgeries for aortic dissection where preexisting peripheral vascular disease and femoral cannulation may be sources of skeletal muscle ischemia. Aortic dissection surgery had a longer CPB and anesthesia time [26, 31]. In our study, patients with RML had significantly longer anesthesia and CPB and aortic cross-clamp times. According to Benedetto et al., longer CPB is associated with a higher incidence of AKI in which RML could be the precipitating factor [6]. Similarly, Conlon et al. reported that longer CPB could be a predictor of AKI [32].

We consider that multiple factors could explain this association in our patients, including prolonged CPB and surgery time. Severe RML triggers a cascade with many consequences, including hypovolemia, hypoalbuminemia, anemia, disseminated intravascular coagulation, hyperkalemia, hypocalcemia, hypercalcemia, hyperphosphatemia, and acute tubular necrosis. Higher pressure on the back muscles related to increased weight and other potential mechanisms related to metabolic derangement are probably present [33] in prolonged surgeries, which are associated with a higher propensity of development of RML in many studies on cardiac and noncardiac surgery [6, 23, 25].

Basal creatinine levels were matched in both groups. While Group I was associated with a significantly high incidence of AKI, it seems that patients with higher levels of CK and myoglobin exhibited a higher association with AKI (Table 2). The incidence of AKI after cardiac surgery is variable. Benedetto et al. found a 2.6-fold increase in the incidence of AKI when myoglobin concentrations reached higher than 465 mg/mL [6]. AKI associated with RML may occur with CK levels as low as 5000 U/L when there is an association of hypovolemia, sepsis, and acidosis. AKI associated with RML usually carries a higher mortality than when RML develops alone (59% versus 22%) [34].

Surgical procedures are currently receiving attention after RML has been linked to trauma that is more pronounced in hypotensive patients. Muscles in the back, as well as the gluteal regions, are compressed against the operating table, leading to RML. This is aggravated by patients' weight, a longer duration of surgery, and CPB-induced hypotension. Peripheral hypoperfusion induced by peripheral vascular disease in CABG patients is hypothesized to exacerbate the condition [6].

Dynamic changes in serum CK and myoglobin levels were studied (Figures 1 and 2), and we observed early diagnostic accuracy for myoglobin with rapid clearance. However, CK levels increased later and were more persistent than myoglobin, which appears to be suitable for follow-up. This finding is consistent with Laurence, who suggested that, as long as the peak of CK had been obtained, estimation of serum CK levels could be helpful for estimating the extent of muscle damage when myocardial infarction is ruled out [35]. Our finding is also consistent with the previous finding that serum myoglobin has faster elimination kinetics in patients treated with forced alkaline diuresis for RML [36].

Our patients were usually placed under sedation after surgery for at least 4 h and received postoperative analgesia. Moreover, pain encountered from a sternotomy incision could mask possible pain from the back. Therefore, early clinical signs for RML were difficult for diagnosis. A high index of suspicion was needed for RML because early prevention is sensible before renal dysfunction is imminent. Six patients in the RML group (35.2%) developed early, unexplained hyperkalemia. Rosenberry et al. mentioned the concomitance of RML and severe hyperkalemia [37]. Receiving sedation and analgesia as well as vasoactive drugs after cardiac surgery could be associated with loss of early clinical signs of RML including fever, malaise, tachycardia, nausea, and vomiting [8].

Patients with RML had a prolonged length of stay in hospital and in the ICU, as well as a prolonged length of mechanical ventilation. We believe that early diagnosis is important for preventing the sequelae of RML. Omar and Abouelnagah suggested that a preventive bundle for RML in bariatric surgery should include adequate padding of pressure points in the preoperative and postoperative periods, proper positioning, with close exposure of the pressure points, reducing the operative time, adequate hydration, and close postoperative monitoring [38]. We think that prevention of RML after cardiac surgery could start with early monitoring.

## 5. Conclusions

An early elevation of serum CK above 2500 U/L postoperatively in high-risk cardiac surgery could be used to diagnose RML that may predict the concomitance of early AKI. Hyperkalemia may be an early warning sign for RML development. Proper intervention may prevent the sequelae of organ dysfunction.

## Figures and Tables

**Table 1:** Clinical variables in both groups.

Variable	Group I (RML) *N* = 17	Group II (no RML) *N* = 184	*P* value
Age	47 ± 11	52 ± 11	0.08

Sex male	16 (94.1%)	169 (91.8%)	0.74

Hypertension	14 (82.5%)	123 (66.8%)	0.15

IDDM	0	16	0.28
NIDDM	4	79	0.07

BMI	28.7 ± 5.9	27.4 ± 4.8	0.4

Ethnicity (Arab)	4 (23.5%)	56 (30.4%)	0.43

ASA III	7 (41.1)	88 (47.8)	0.7
ASA IV	10 (58.8)	96 (52.1)	0.6

Euro score	3 ± 2.5	2.5 ± 2.3	0.47

Liver diseases	1 (5.8%)	2 (1%)	0.23

CRD	3 (17.6%)	18 (9.6%)	0.25

Statin usage	8 (47.1)	136 (73.5)	0.02

Inotrops			
Dopamine	10 (58.8%)	47 (25.5%)	0.03
Adrenaline	3 (17.6%)	24 (13%)	0.4
Noradrenline	6 (35.3)	52 (28.4%)	0.3

Surgery			
CABG	12 (70.6%)	140 (76%)	0.6
Valvular	2 (11.7%)	40 (21.7%)	0.01
Aortic dissec.	3 (17.7%)	0	0.001

Procedure type (urgent)	5 (29.4%)	41 (22.2%)	0.59

CPB time (minutes)	181.7 ± 75	110.7 ± 47	0.004

ACC time (minutes)	110.2 ± 12	68.6 ± 2.4	0.0001

Anesthesia time (min)	478.5 ± 168	362.7 ± 25	0.017

IABP	1 (5.8%)	9 (4.8%)	0.7

CHF	6 (35.3%)	66 (35%)	0.6

Complication			
Arrhythmia	3 (17%)	17 (7.5%)	0.2
MI	0	9 (4.9%)	0.4
Infection	2 (11.8%)	4 (2.2%)	0.05
Early stroke	1	0	

IDDM: insulin-dependant diabetes mellitus; NIDDM: non-insulin-dependent diabetes mellitus; BMI: body mass index; CBG: coronary artery bypass graft; CPB: cardiopulmonary bypass; ACC: aortic cross-clamp; IABP: intra-aortic balloon pump; CHF: congestive heart failure; MI: myocardial infarction.

**Table 2:** Laboratory and prognostic variables in both groups.

Variable	Group I (RML) *N* = 17	Group II (no RML) *N* = 184	*P* value
Basal creatinine (micromole/L)	95.6 ± 35.3	92.7 ± 61	0.8
AKI	7 (41%)	34 (18%)	0.025
HsTnT (ng/L)	3606 ± 110	1064 ± 81	0.001
CK MB (U/L)	83.4 ± 26	40.3 ± 3.1	0.001
Myoglobin (ng/mL)	1120 ± 250	450 ± 195	0.0001
LOS_ICU_ (hours)	203 ± 142	50 ± 11	0.001
LOS_hosp_ (days)	13.06 ± 11	7.5 ± 3.7	0.001
Ventilation time (minutes)	856 ± 199	486 ± 24	0.001

AKI: acute kidney injury; HsTnT: high sensitive troponin T; CK MB: creatine kinase MB; LOS_ICU_: length of stay in intensive care; LOS_hosp_: hospital length of stay.

**Table 3:** Multivariate logistic regression analysis for CK above 2500.

Variable	Adjusted OR	95% CI	Significance
Age	0.934	.852–1.023	0.934
Anesthesia time	2.02	.994–1.004	0.7
CPB time		.976–1.023	0.85
ACC time		.999–1.071	0.06
Surgery			
CABG	0.72	0.002–0.73	0.03
Valvular	1.34	0.019–20.6	0.7
Dopamine usage	0.8	.034–.898	0.037
LOV (minutes)	1	.998–1.002	0.8
LOS_ICU_ (hours)	.99	0.998–1.003	0.8
LOS_hosp_ (days)	.99	0.954–1.174	0.2
AKI	0.37	0.073–2.962	0.08

CPB: cardiopulmonary bypass; ACC: aortic cross clamp; CABG: coronary artery bypass graft; LOV: length of mechanical ventilation; LOS_ICU_: ICU length of stay; LOS_hosp_: hospital length of stay; AKI: acute kidney injury.

**Table 4:** CK and myoglobin relation.

Variable	*r*	*P*
CK & myoglobin	0.63	0.001

CK: creatine kinase.

## Abbreviations

ASA: American Society of Anesthesiologists
AKI: Acute kidney injury
CABG: Coronary artery bypass grafting
CAD: Coronary artery disease
CK: Creatine kinase
CPB: Cardiopulmonary bypass
IABP: Intra-aortic balloon pump
RML: Rhabdomyolysis.
